# RET inhibition overcomes resistance to combined CDK4/6 inhibitor and endocrine therapy in ER+ breast cancer

**DOI:** 10.3389/fonc.2024.1497093

**Published:** 2025-01-27

**Authors:** Charlotte K. Kindt, Sidse Ehmsen, Sofie Traynor, Benedetta Policastro, Nikoline Nissen, Mie K. Jakobsen, Monique F. Hundebøl, Lene E. Johansen, Martin Bak, Elsa Arbajian, Johan Staaf, Henrik J. Ditzel, Carla L. Alves

**Affiliations:** ^1^ Department of Cancer and Inflammation Research, Institute of Molecular Medicine, University of Southern Denmark, Odense, Denmark; ^2^ Department of Oncology, Odense University Hospital, Odense, Denmark; ^3^ Institute of Clinical Research, University of Southern Denmark, Odense, Denmark; ^4^ Department of Pathology, Sydvestjysk Sygehus, Esbjerg, Denmark; ^5^ Division of Oncology, Department of Clinical Sciences Lund, Lund University, Lund, Sweden

**Keywords:** estrogen receptor-positive breast cancer, RET, selpercatinib, CDK4/6 inhibitor, drug resistance

## Abstract

**Background:**

Combined CDK4/6 inhibitor (CDK4/6i) and endocrine therapy significantly improve the outcome of patients with advanced estrogen receptor-positive (ER+) breast cancer. However, resistance to this treatment and disease progression remains a major clinical challenge. High expression of the receptor tyrosine kinase REarranged during Transfection (RET) has been associated with resistance to endocrine therapy in breast cancer, but the role of RET in CDK4/6i treatment response/resistance remains unexplored.

**Methods:**

To identify gene expression alterations associated with resistance to combined endocrine therapy and CDK4/6i, we performed RNA sequencing of two ER+ breast cancer cell models resistant to this combined therapy. The functional role of RET was assessed by siRNA-mediated *RET* silencing and targeted inhibition with the FDA/EMA-approved RET-selective inhibitor selpercatinib in resistant breast cancer cells and patient-derived organoids (PDOs). *RET* silencing was evaluated mechanistically using global gene expression and pathway analysis. The clinical relevance of RET expression in ER+ breast cancer was investigated by gene array analysis of primary tumors treated with endocrine therapy and by immunohistochemical scoring of metastatic lesions from patients who received combined CDK4/6i and endocrine therapy.

**Results:**

We show that RET is upregulated in ER+ breast cancer cell lines resistant to combined CDK4/6i and fulvestrant compared to isogenic cells resistant to fulvestrant alone. siRNA-mediated silence of *RET* in high RET-expressing, combined CDK4/6i- and fulvestrant-resistant cells reduced their growth partially by affecting cell cycle regulators of the G2-M phase and E2F targets. Notably, targeting RET with selpercatinib in combination with CDK4/6i inhibited the growth of CDK4/6i-resistant cell lines and resensitized ER+ breast cancer patient-derived organoids resistant to CDK4/6i. Finally, analysis of RET expression in ER+ breast cancer patients treated with endocrine therapy showed that high RET expression correlated with poor clinical outcomes. We further observed a shorter median survival to combined CDK4/6i and endocrine therapy in patients with RET-positive compared to RET-negative tumors, but this difference did not reach statistical significance.

**Conclusions:**

Our findings show that RET is overexpressed in ER+ metastatic breast cancer resistant to combined CDK4/6i and endocrine therapy, rendering RET inhibition a promising therapeutic approach for patients who experience disease progression on combined CDK4/6i and endocrine therapy.

## Introduction

Estrogen receptor-positive (ER+) breast cancer comprises approximately 70% of all breast cancers and is dependent on the ER pathway for proliferation and survival. Therefore, inhibition of the ER pathway is an effective treatment strategy in this patient population (1). However, resistance to endocrine treatment remains a major clinical challenge (2). Several studies have shown that endocrine resistance mechanisms depend on alterations of cell cycle regulators, which led to the development of cyclin-dependent kinases 4 and 6 inhibitors (CDK4/6i) for ER+ advanced breast cancer (3–6).

CDK4/6 are critical regulators of the G1-S phase progression in the cell cycle by interacting with cyclin D and subsequently hyperphosphorylating the retinoblastoma (Rb) protein (7). This leads to its inactivation and release of transcription factors that allow progression to the cell cycle S-phase. CDK4/6i, including ribociclib, palbociclib, and abemaciclib, inhibit the CDK4/6 kinases and thus arrest the cells in the G1 phase. Clinical studies have shown that adding CDK4/6i to endocrine therapy improved progression-free survival (PFS) and overall survival (OS) compared to endocrine therapy alone in patients with ER+ advanced breast cancer. This resulted in the approval of combined CDK4/6i and an aromatase inhibitor (AI) for ER+ advanced breast cancer as first-line treatment (1, 8, 9) and as second-line therapy in combination with the selective estrogen-receptor degrader (SERD) fulvestrant following initial AI monotherapy (10–12). More recently, the CDK4/6i abemaciclib was also approved for high-risk patients with early-stage ER+ breast cancer in combination with tamoxifen or an AI (13). Despite favorable outcomes, the development of resistance to combined CDK4/6i and endocrine therapy is expected in the metastatic setting, and 70% of these patients will experience progressive disease within 40 months (14, 15). Understanding resistance mechanisms to combined CDK4/6i and endocrine therapy and identifying optimal treatment strategies following progression on combined CDK4/6i and endocrine therapy are currently areas of intense research.

In breast cancer, increased levels of the receptor tyrosine kinase REarranged during Transfection (RET) proto-oncogene have been observed in tumors compared to surrounding healthy tissue, and high RET expression has been associated with tamoxifen and AI resistance in ER+ breast cancers (16–18). RET comprises an extracellular domain, a cysteine-rich region, a single-pass transmembrane domain, and a cytoplasmic region with a split tyrosine kinase domain. The binding of RET to its ligands requires the glial cell-derived neurotrophic factor (GDNF) receptor α family (GFRα 1-4) coreceptors. GFRα forms homodimers recruited by specific GDNF family of ligands (GFLs) into a complex that activates RET homodimers, leading to autophosphorylation of the tyrosine kinase domain (19). RET activation initiates the activation of the MAPK/ERK, JAK/STAT, and PI3K/AKT pathways, leading to proliferation, survival, and migration (20, 21).

Furthermore, RET expression correlates with ER expression in breast cancer cell lines and tumor specimens (22). Multiple studies have shown that ER induces the expression of RET, which leads to the activation of downstream growth signaling pathways. Conversely, RET has been shown to enhance estrogen-mediated proliferation (22, 23). Overexpression of RET alone has been shown to increase growth of ER+ breast cancers in mice (24). Importantly, targeting RET with the multikinase inhibitor vandetanib potentiated the effect of tamoxifen, demonstrating a greater reduction in tumor growth compared to single-agent therapy (25). Furthermore, the RET inhibitor NVP-AST487, in combination with the AI letrozole, was also shown to be effective in inhibiting breast cancer cell line motility and growth (26). Notably, RET activation promotes AI and tamoxifen resistance through estrogen-independent activation of ER transcriptional activity via the MAPK/ERK and PI3K/AKT pathways, where mTOR might play a key role (16, 17).

In papillary thyroid carcinoma (PTC) and non-small cell lung cancer (NSCLC), RET fusion proteins that are constitutively active and promote tumor growth have been identified in 13-43% and 2% of patients, respectively (27–29). In addition, up to 70% of medullary thyroid cancers (MTC) show activating *RET* mutations, but *RET* fusions and mutations are rare in breast cancer (30–32). The European Medicines Agency (EMA) and the Food and Drug Agency (FDA) have recently approved the RET-selective inhibitor (RETi) selpercatinib in *RET* fusion-positive advanced NSCLC and PTC and *RET*-mutant MTC (33, 34). More recently, the FDA granted accelerated approval to selpercatinib as a tissue-agnostic treatment of locally advanced or metastatic solid tumors with *RET* gene fusions after prior systemic treatment, or without alternative treatment options (35). Selpercatinib is effective towards RET-wildtype, -mutant, and -fusion proteins (36). Although RET has been associated with ER+ breast cancer tumorigenesis and endocrine treatment response (22, 23, 25), the role of RET in resistance mechanisms to combined fulvestrant and CDK4/6i has not been evaluated.

This study shows that RET overexpression is associated with resistance to combined CDK4/6i and fulvestrant treatment in ER+ breast cancer cell lines. Inhibition of RET by siRNA-mediated knockdown or treatment with the tyrosine kinase inhibitor selpercatinib impaired the growth of CDK4/6i- and fulvestrant-resistant cell lines and patient-derived organoids by inhibiting, at least in part, early stages of mitotic cell division. Finally, we show that clinical ER+ breast cancer samples expressing high mRNA levels of *RET* correlated with poor clinical outcomes following endocrine therapy.

## Methods

### Cell lines and anti-tumor agents

The original MCF7 (RRID: CVCL_0031), T47D (RRID: CVCL_0553), and ZR-75-1 (RRID: CVCL_0588) cells were received from the Breast Cancer Task Force Cell Culture Bank, Mason Research Institute. The MCF-7-derived cell lines MF-R and MPF-R were developed by extended treatment with fulvestrant (100 nM) alone or combined with CDK4/6i (palbociclib, 150-200 nM), respectively. Cells grew in Dulbecco’s Modified Eagle Medium phenol red-free (DMEM/F12; Gibco) with 1% glutamine (Gibco), 1% heat-inactivated fetal bovine serum (FBS; Sigma-Aldrich), and 6 ng/mL insulin (Sigma-Aldrich) supplemented with 100 nM of fulvestrant and 200 nM CDK4/6i. MCF-7 cells grown in parallel with MPF-R cells without treatment in the media were designated M-S and remained sensitive to drug treatment. T47D cells were maintained in the Roswell Park Memorial Institute (RPMI) 1640 medium (Gibco) without phenol red, supplemented with 1% glutamine, 5% heat-inactivated FBS, and 8 µg/mL insulin. The T47D-derived fulvestrant- and combined CDK4/6i- and fulvestrant-resistant cell lines, TF-R and TPF-R, respectively, were established by long-term treatment with 100 nM fulvestrant alone or combined with 150-200 nM of CDK4/6i. T47D-sensitive cells grown in parallel with TPF-R were designated T-S and were maintained in the same medium as TPF-R cells without treatment. ZR-75-1 cells resistant to combined fulvestrant and palbociclib (ZPF-R) were established from fulvestrant-resistant cells (37) by long-term treatment (2 months) with 100 nM fulvestrant and increasing concentrations of palbociclib (weekly 2-fold increase, from 10 nM to 160 nM). Cells were cultured at 37°C in 5% CO_2_ and tested regularly for mycoplasma with a MycoAlert detection kit (Lonza), and all experiments were performed with mycoplasma-free cells. Cells underwent authentication by short tandem repeat (STR) analysis in the past three years. Fulvestrant (ICI 182,780, Tocris) was dissolved in 96% ethanol, CDK4/6i palbociclib isothiocyanate (HY-A0065, MedChemExpress) was dissolved in water, RET inhibitor selpercatinib (also known as LOXO-292, HY-114370, MedChemExpress) was dissolved in DMSO (Sigma-Aldrich). The concentrations of CDK4/6i and RET inhibitor used for *in vitro* experiments were determined based on the IC50 for each cell line model.

### Patient-derived organoid studies

Organoids were established from ER+ primary tumors as previously described [15]. Briefly, tumors were mechanically dissociated in a gentleMACS dissociator (Miltenyi Biotec) and enzymatically digested with 1 mg/mL collagenase (Thermofisher) for 1 hour at 37°C. After dissociation, cells were embedded in cultrex (Bio-techne) and plated in 15 µL domes in six-well cell culture plates. Plates were incubated for 30 minutes at 37°C for solidification of the matrix and then, organoid medium consisting of advanced DMEM/F12 (ThermoFisher) supplemented with 10 mM HEPES (Gibco), 1× Glutamax (Gibco), 1% penicillin/streptomycin (ThermoFisher), 2% Rspo3-Fc fusion protein conditioned medium (Ipatherapeutics), 1% Noggin-Fc Fusion Protein Conditioned Medium (Ipatherapeutics), 1x B27 Supplement (ThermoFisher), 5 mM Nicotinamide (Sigma Aldrich), 1.25 mM N-acetyl-ʟ-cysteine (Sigma Aldrich), 100 µg/mL Primocin (Invivogen), 5nM Heregulin β1 (PeproTech), 5 ng/mL FGF-7 (PeproTech), 10 ng/mL heat-stable FGF-10 (ThermoFisher), 0.5 µM A83-01 (Tocris Bioscience), 10 ng/mL EGF (PeproTech), 0.5 µM SB202190 (Sigma Aldrich) and 5µM Y-27632 dihydrochloride (AbMole Bioscience) was added. Media were renewed every 3 to 4 days. Organoids were dissociated using TrypleExpress (ThermoFisher) for 15 minutes. Before the described experiments, established organoids were tested for mycoplasma (MycoAlert, Lonza).

For drug testing, organoids were plated in 96-well plates at a concentration of 200 organoids/µL in cultrex (50 µL/well), followed by treatment with serially diluted CDK4/6i palbociclib, RETi selpercatinib or both, at different concentrations for 7 days. Cell viability was evaluated with RealTime-Glo™ MT cell viability assay (Promega) according to the manufacturer’s instructions. A Paradigm microplate reader (Beckman Coulter) and SoftMax pro 7.0.2 software were used to measure luminescence. Dose-effect curves were generated using GraphPad Prism software (version 9.0). Data was inputted into SynergyFinder (38) to calculate the Bliss synergy score.

### Western blotting

Whole-cell extracts were obtained using RIPA buffer (50 mM Tris HCl (pH 8), 150 mM NaCl (pH 8), 1% IgePAL 630, 0.5% sodium deoxycholate, 0.1% SDS) with protease and phosphatase inhibitors (Thermo Scientific). Pierce BCA Protein Assay kit (Thermo Scientific) was used to determine protein concentration at 562 nm in the Paradigm microplate reader (Beckman Coulter). Protein (10-45 µg) was loaded on a 4-20% SDS-PAGE gel (Bio-Rad) under reducing conditions and electroblotted onto a PVDF transfer membrane (Bio-Rad). Tris-buffered Saline (TBS) with 0.1% Tween-20 (Sigma-Aldrich) and 5% non-fat dry milk powder (Sigma-Aldrich) was used to block membranes for one hour at room temperature. The following antibodies were used: Anti-RET (3223S, 1:250-1:1000), anti-phospho-Rb Ser780 (3590, 1:1000), anti-Rb (9309, 1:1000), anti-cyclin D1 (2978, 1:1000), anti-phospho-CDK2 Thr160 (2561, 1:500), anti-cyclin E2 (4132, 1:1000), anti-CDK2 (2546, 1:2000), anti-cyclin D3 (3223, 1:2000) and anti-cyclin A (4656, 1:2000) from Cell Signaling Technologies; anti-cyclin A2 (ab38, 1:500) and anti-CDK6 (ab12482, 1:5000) from Abcam; anti-cyclin E1 (sc-247, 1:100), anti-CDK4 (sc-23896), and anti-GAPDH (sc-32233, 1:20000) from Santa Cruz Biotechnology. Horseradish peroxidase (HRP)-conjugated goat anti-mouse (P0447, Dako, 1:5000) and HRP-conjugated goat anti-rabbit (P0448, Dako, 1:5000) antibodies were incubated in blocking buffer for one hour at room temperature. Membranes were developed with SuperSignal™ West Pico PLUS chemiluminescent Substrate (Thermo Scientific) and visualized on a ChemiDoc MP imaging system (Bio-Rad).

### RET-specific siRNA-mediated knockdown

*RET* gene knockdown was performed using two different RET-specific siRNAs (RET_15, SI04950554 and RET_17, SI05089756), both from Qiagen and a nontargeting scrambled (control) siRNA used as the universal negative control (SIC001, Sigma-Aldrich). Chemical transfection was performed in M-S, MPF-R, T-S, and TPF-R cell lines with Lipofectamine 3000 transfection reagent (15282465, ThermoFisher Scientific) in Opti-MEM medium (Gibco) according to the manufacturer’s instructions. Efficiency was evaluated at the mRNA level 48 hours after transfection by quantitative real-time PCR (RT-qPCR) and at the protein level 96 hours after transfection with Western blotting. The effect of siRNA-mediated knockdown of RET on cell growth was evaluated with crystal violet assay at 24, 48, 96, and 144 hours after transfection.

### RNA extraction, cDNA synthesis, quantitative real-time PCR

TRI reagent^®^ (Sigma Aldrich) was used for total RNA extraction, and cDNA synthesis was performed with random deoxynucleic acid hexamers and reverse transcriptase (Fermentas). RT-qPCR with SYBR Green PCR Mastermix (Applied Biosystems) was performed according to the manufacturer’s instructions. The following primers (Qiagen) were used: *RET* (QT00047985, transcript ID: ENST00000355710, amplicon length 120), CDK6 (QT00019985, transcript ID: ENST00000265734, amplicon length 82), CDK4 (QT00016107, transcript ID: ENST00000257904, amplicon length 60) and cyclin D1 (QT00495285, transcript ID: ENST00000227507, amplicon length 96), and *PUM1* (QT00029421, transcript ID: ENST00000257075, amplicon length 73) was used as a reference gene. The RT-qPCR reactions were performed using a StepOnePlus system (Applied Biosystems), and data were analyzed with StepOne Software. Reactions were conducted in triplicates, and data were analyzed with the delta-delta CT method (39).

### Global gene expression analysis

Gene expression analysis was performed on RNA purified from three biological replicates of the combined CDK4/6i- and fulvestrant-resistant cell lines MPF-R and TPF-R treated with control or *RET* siRNAs using Human Transcriptome Arrays 2.0 (HTA). Cells were grown to reach 70-80% confluency, and RNA was purified using TRI reagent® according to the manufacturer’s instructions. Transcriptome Analysis Console (TAC) software (ThermoFisher) was used for data analysis. Genes from the dataset that exhibited a two-fold or greater alteration in expression with a false discovery rate (FDR) < 0.05 cut-off and *p* < 0.05 with one-way ANOVA were considered significantly regulated. Gene Set Enrichment Analysis (GSEA 4.3.2) was performed to identify the gene sets enriched in the resistant cells. Microarray gene expression data from the tumor biopsies in the NeoPalAna trial (40) was used to investigate *RET* expression (GSE93204). Normalized *RET* expression data from baseline samples from CDK4/6i-sensitive or -resistant patients were used for the analysis.

### RNA sequencing

Exon-spanning primers were designed to perform RNA sequencing (primer sequences are available upon request). RNA from three independent experiments were prepared for sequencing on the Illumina NovaSeq 6000 platform using the NEBNext Poly(A) mRNA Magnetic Isolation Module (New England Biolabs) and the NEBNext Ultra II DNA Library Prep Kit for Illumina (New England Biolabs) with unique dual indexes according to the manufacturer’s instructions. FASTQC (Babraham Bioinformatics) was used to assess the quality of raw sequencing reads, and adaptor sequences were removed using the FASTX toolkit. Trimmed Reads were aligned to the human genome (hg38) using the Spliced Transcripts Alignment to a Reference (STAR) software with default parameters (41). Tags in exons were counted using iRNA-seq (42), and differential expression (FDR-adjusted *p <* 0.05) between three independent replicates of sensitive cell line and double-resistant cell line samples was determined using DESeq2 (43). Genes with FDR ≤ 0.05 and a log2 fold change > 1.0 in either direction were defined as statistically significant. GSEA and gene ontology (GO) enrichment analysis with ShinyGO (v0.80) were used to identify the gene sets enriched in cells resistant to combined CDK4/6i and fulvestrant versus fulvestrant alone. To identify candidate fusion transcripts from the sequence data, fusion calling was performed on the fastq files using FusionCatcher version 1.33 (44), STAR-fusion version 1.11.0 (45), and Arriba version 2.3.0 (46), with default settings. The GRCh38/hg38 build was used as the human reference genome.

### Cell growth assay

Cells were seeded at 20,000-50,000 cells/well in 96-well plates, and drugs or vehicles were added after 24 hours. Cell growth was evaluated using a crystal violet-based colorimetric assay wherein cells were incubated with a crystal violet solution for 5 minutes, followed by three washes in ddH2O and overnight drying. Cellular crystal violet was extracted by incubation with a 0.1 M citrate buffer (29.41 g sodium citrate dissolved in 50% water and 50% ethanol, pH=6) for 30 minutes on a shaker at room temperature. Reading measurements were performed in a Paradigm microplate reader at 570 nm and data were analyzed with SoftMax pro 7.0.2 software.

### KM plotter

The web tool Kaplan-Meier (KM) plotter (47) was used to generate survival curves for ER+ breast cancer patients based on mRNA expression (gene chip) of *RET*. All datasets available in the KM plotter were included in the analysis. The inclusion criteria were ER status positive by IHC, HER2 status negative by array, and previous treatment with endocrine therapy. These criteria were independent of pathological characteristics such as grade, lymph node status, and previous chemotherapy. The JetSet optimal probe was selected for *RET* (probe ID 211,421). The ‘auto select best cutoff’ option was used to evaluate all possible cutoff values between the lower and upper quartiles, and the best performing threshold was selected as a cutoff (**Supplementary Table S1**, selected cutoff highlighted). The endpoints were relapse-free survival (RFS) and overall survival (OS).

### Clinical samples and endpoints

Formalin-fixed, paraffin-embedded (FFPE) ER+ metastatic tumor lesions from patients treated with combined CDK4/6i and endocrine therapy were obtained from Odense University Hospital (OUH) (N = 115). Inclusion criteria were patients with ER+ advanced breast cancer treated with combined CDK4/6i and endocrine therapy in the metastatic setting who had undergone surgery or biopsy at OUH. Tumor sections with ER expression ≥ 1% were considered ER+. Exclusion criteria were insufficient tumor material and no metastatic biopsy before starting treatment with combined CDK4/6i (palbociclib or ribociclib) and endocrine therapy (letrozole or fulvestrant). These criteria yielded N = 83 patients. Progression-free survival (PFS) was described as the time from starting treatment with combined CDK4/6i and endocrine therapy until disease progression or death.

### Immunohistochemistry

FFPE blocks of patient metastatic lesions were sectioned at 4 µm with a microtome and mounted on ChemMateTM Capillary GAP slides (Dako). Sections were dried at 60°C, deparaffinized, hydrated, and endogenous activity was blocked. Unmasking of epitopes was achieved by boiling sections in a T-EG solution. The following primary antibody was used: anti-RET antibody (ab134100, 1:50). Primary antibody binding for anti-RET was detected with Optiview-DAB (8–8), EnV, FLEX/HRB+ Rabbit LINK 15-30. An experienced breast pathologist evaluated the clinical samples in a blinded setup. RET protein was primarily expressed in the cytoplasm. The staining intensity was recorded on a semi-quantitative scale of 0-3, with 0 meaning absolutely no reaction and 3 as the most intense staining. The cut-off value for positive versus negative was set as intensity ≥ 1 versus intensity = 0 and determined based on the survival significance.

### Statistical analysis

GraphPad Prism v.9.4.0 software was used for statistical analyses. One-way analysis of variance (ANOVA) and two-tailed t-test were performed to determine statistical significance among data for the *in vitro* studies (as indicated in the figure legends). Kaplan-Meier estimates generated survival curves for the clinical data, and the log-rank test was used to test the correlation between the expression levels of RET and the PFS. *p-*values were defined as follows: *p<0.05, **p<0.01, *** p<0.001, and **** p<0.0001.

## Results

### RET is upregulated in ER+ breast cancer cells resistant to combined CDK4/6i and fulvestrant

**Figure 1:**
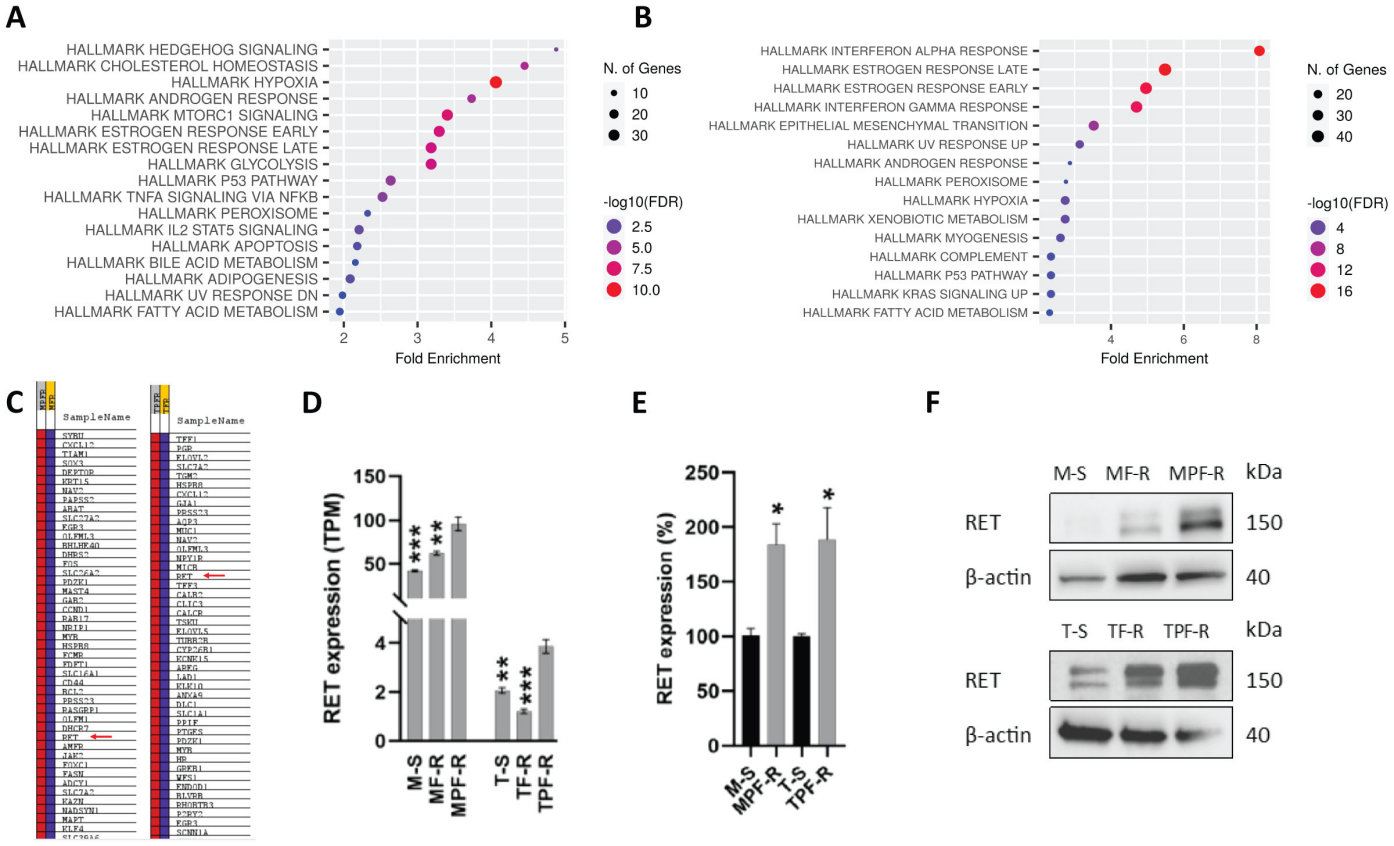
RET is overexpressed in ER+ breast cancer cell lines resistant to combined CDK4/6i and endocrine therapy. Dot plots of Hallmark gene set significantly enriched in **(A)** MPF-R vs. MF-R and **(B)** TPF-R vs. TF-R. **(C)** List of genes enriched in gene set “Hallmark estrogen response early”. **(D)** Evaluation of RET expression in ER+ breast cancer cell lines resistant to combined palbociclib and fulvestrant (MPF-R and TPF-R), resistant to fulvestrant only (MF-R and TF-R), and parental sensitive cells (M-S and T-S) using RNA sequencing. Statistical comparison is shown relative to double-resistant cells. TPM = transcripts per million. The data represent independent experiments in triplicates ± SEM. **(E)** Quantitative RT-PCR verifying the gene expression alterations of *RET*. The expression was normalized using the *PUM1* gene and shown as a relative expression in MPF-R vs. M-S and TPF-R vs T-S cells. Data represent three independent experiments ± SEM (*0.01 < p < 0.05). **(F)** Western blotting analysis of lysates from M-S, MF-R, MPF-R, T-S, TF-R and TPF-R cells. 10 µg and 50 µg of total protein of MCF-7- and T47D-derived cells, respectively, were loaded. β-actin was used as a loading control. A representative for three biological replicates is shown. Asterisk indicate significant differences in students t-test (*0.01 < p < 0.05, **0.001 < p < 0.01 and ***0.0001< p <0.001).

To investigate the resistance mechanisms to combined CDK4/6i and fulvestrant, we utilized two ER+ breast cancer cell line models resistant to combined CDK4/6i and fulvestrant derived from MCF7 and T47D cells (MPF-R and TPF-R, respectively), which have been previously described (37, 48). To identify the resistance mechanism associated specifically with combined CDK4/6i and fulvestrant rather than fulvestrant alone, we evaluated gene expression alterations in MPF-R and TPF-R cells compared to the respective cell lines resistant to fulvestrant alone (MF-R and TF-R). We focused on this comparison instead of comparison with drug-sensitive cells (M-S and T-S) since we aimed to identify gene alterations uniquely associated with resistance to the combination therapy and distinct from those caused by endocrine therapy alone. Using RNA-sequencing, we identified a total of 1103 genes (523 upregulated and 580 downregulated) that exhibited significantly altered expression in MPF-R versus MF-R, and 1041 genes (600 upregulated and 441 downregulated) that exhibited significantly altered expression in TPF-R versus TF-R cells (fold-change ≥ 2, FDR < 0.05, Wald significance test *p <* 0.05). Gene set enrichment analysis (GSEA) was performed to identify the most altered pathways in combined CDK4/6i and fulvestrant-resistant vs. fulvestrant-resistant cells. The enrichment analysis revealed that the top hallmark pathways altered in both MPF-R vs. MFR and TPF-R vs. TFR included “estrogen response early” and “estrogen response late” (**Figures 1A, B**). Although *RET* ranked 34^th^ and 15^th^ in the list of genes enriched in the gene set “estrogen response” in MPF-R and TPF-R cells, respectively (**Figure 1C**), only a few genes were shared between the two models, with *RET* consistently being in the top 5 of the common genes. Notably, neither model showed an alteration of the RET ligand *GDNF* between resistant and sensitive cells. These findings, together with the established role of RET in ER+ breast cancer, prompted us to further investigate *RET* in the context of resistance to combined CDK4/6i and endocrine therapy. Increased expression of *RET* was also observed in MPF-R and TPF-R compared to M-S and T-S cells, respectively (**Figure 1D**). Notably, *RET* expression levels were significantly higher in the MCF7-derived cells than in the T47D-derived cells, as previously reported (16), which suggests a more critical role for RET in MCF-7 cells. Next, we investigated whether *RET*-fusions could be identified in our RNA sequencing data of MPF-R, TPF-R, MF-R, TF-R, M-S, and T-S cells. However, no fusion transcripts involving the *RET* gene were identified in any of the ER+ breast cancer cell lines using three fusion callers (Fusioncatcher, STAR-fusion, and Arriba). Notably, combined fulvestrant and CDK4/6i-resistant cells do not exhibit *RET* mutations or alterations associated with known/putative mechanisms of resistance towards CDK4/6i, such as *ESR1* mutations or *CDK4* or *CDK6* amplifications (48). To further validate the overexpression of RET in combined CDK4/6i- and fulvestrant-resistant cells, qPCR and Western blotting were performed. Overexpression of RET at the mRNA and protein levels in resistant cells was confirmed (**Figures 1E, F**). Importantly, while RET expression is elevated in fulvestrant-resistant cells compared to sensitive cells, the highest expression levels are observed in combined CDK4/6i- and fulvestrant-resistant cells (**Figure 1F**). Because of the higher levels of RET in the MCF7 model, the amount of protein from T47D-derived cells loaded to detect RET expression was five times that from MPF-R cells (**Figure 1F**). Our findings support a significant upregulation of RET in both cell line models resistant to combined CDK4/6i and fulvestrant.

### *RET*-specific siRNA-mediated knockdown impairs the growth of combined CDK4/6i- and fulvestrant-resistant ER+ breast cancer cells

**Figure 2:**
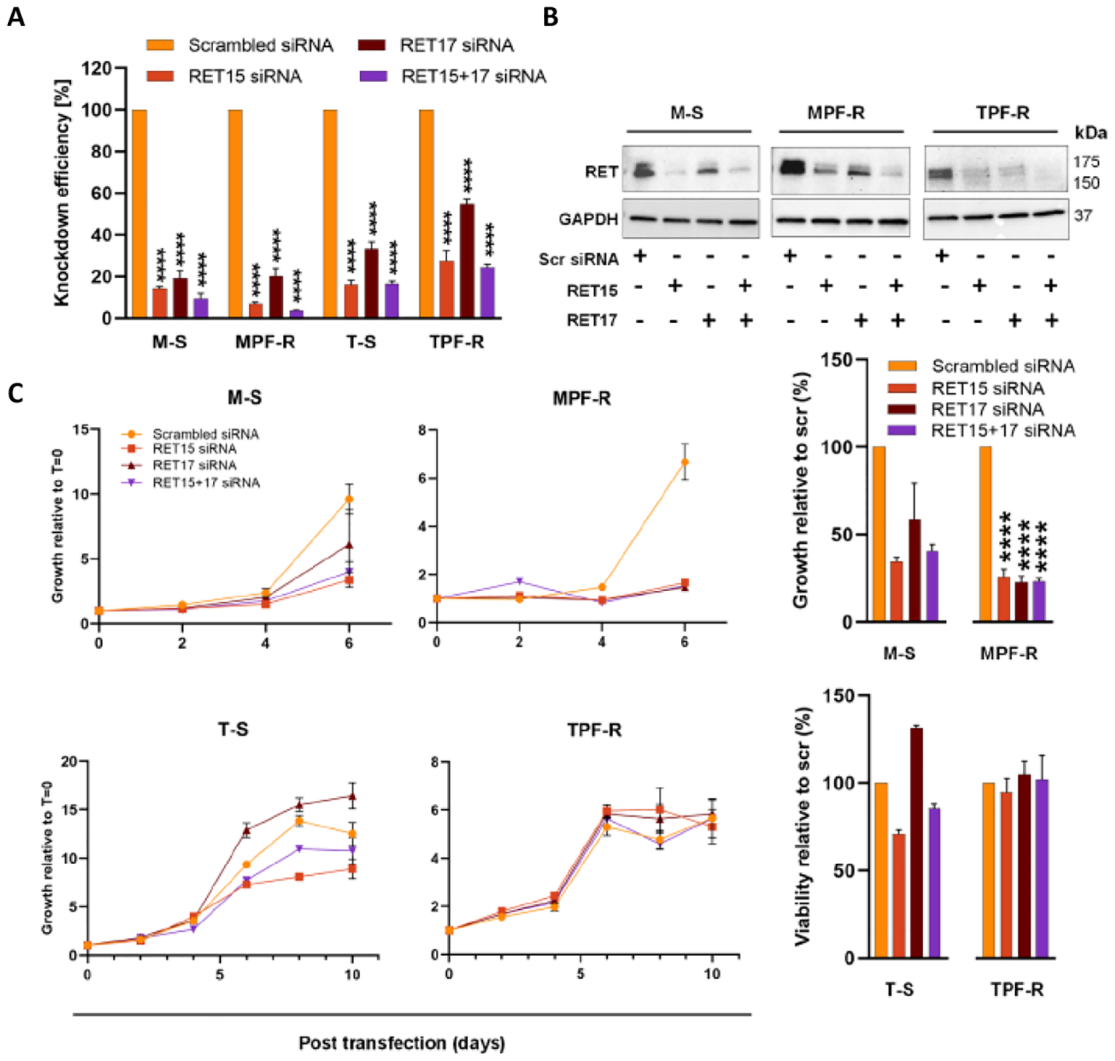
RET-specific siRNA-mediated knockdown inhibits the growth of MPF-R breast cancer cells. The efficiency of *RET* silencing in combined CDK4/6i- and fulvestrant-resistant cell lines (MPF-R and TPF-R) and their parental sensitive cell lines (M-S and T-S), respectively, transfected with two different *RET*-specific siRNAs (RET15 and RET17) or scrambled siRNA (control). **(A)** RT-qPCR verifying reduction of *RET* mRNA level 48 h post-transfection with *RET*-specific siRNA. The expression was normalized using the *PUM1* gene. The knockdown efficiency is represented as the average percentage compared to the control (scr) of triplicates (mean ± SEM). **(B)** Western blot validation of protein levels 96 h post-transfection with *RET*-specific siRNAs. GAPDH was used as a protein loading control. **(C)** Cell growth at different time points following *RET*-specific siRNA transfection as assessed by crystal violet assay. Graph columns show cell growth at days 6 and 10 for MCF7-derived cell lines and T47D-derived cell lines, respectively. Scrambled siRNA: control siRNA; RET15, and RET17: two different *RET*-specific siRNAs. RET15 + 17: combination of both *RET*-specific siRNAs. Asterisks indicate significant differences in the one-way ANOVA test (****p < 0.0001).

To investigate RET’s role in the resistance mechanism to combined CDK4/6i and fulvestrant, we performed gene knockdown by using two specific siRNAs targeting *RET* (RET15 and RET17) and a scrambled siRNA (control). *RET* was efficiently silenced in both MCF-7- and T47D-derived sensitive and resistant cell lines when using the individual and pooled *RET*-siRNAs compared with the control siRNA, as determined by RT-qPCR and Western blotting (**Figures 2A, B**). It was not possible to visualize the RET knockdown in T-S cells by Western blotting due to the extremely low levels of RET in these cells. Silencing of RET significantly reduced the growth of M-S and MPF-R cells compared to the control siRNA (**Figure 2C**), indicating that these cells depend on the expression of RET for proliferation and growth. The same effect on cell growth upon *RET* siRNA-mediated knockdown was not observed in T-S and TPF-R cells, which may be due to the significantly lower level of *RET* in these cells.

**Figure 3:**
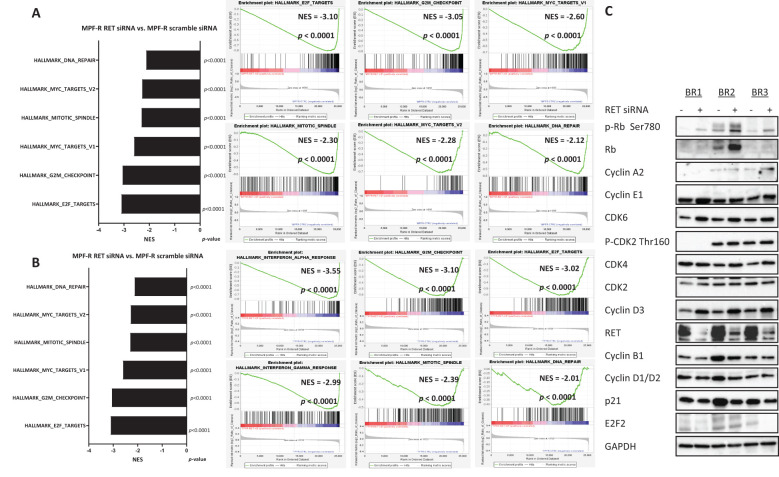
*RET* gene knockdown impairs cell growth of combined CDK4/6i- and fulvestrant-resistant breast cancer cells by blocking the G2-M phase progression of the cell cycle. Bar graphs and enrichment plots of Hallmark gene sets significantly enriched in **(A)** MPF-R *RET*-siRNA vs. control-siRNA and **(B)** TPF-R *RET*-siRNA vs. control-siRNA. *RET*-siRNA 15 and RET-siRNA 17 pools were used. Statistical significance (nominal P-value) of the enrichment score (ES) is calculated using an empirical phenotype-based permutation test. **(C)** Western blotting of cell cycle regulators in three biological replicates (BR) of MPF-R *RET*-siRNA versus control-siRNA. GAPDH was used as a loading control.

To identify pathways altered following *RET* silencing, we performed gene expression analysis on MPF-R and TPF-R cells transfected with *RET*-siRNA (pooled RET15 and RET17) compared to cells transfected with control siRNA (scrambled). Remarkably, alterations in regulators of the cell cycle, particularly regulators of the G2-M phase and E2F targets, were identified as the most significantly enriched gene datasets by GSEA in control-siRNA compared to *RET*-siRNA treated MPF-R and TPF-R cells (**Figures 3A, B**, respectively). In both MCF7- and T47D-derived resistant cells, *RET* knockdown significantly correlated with reduced gene expression of regulators of late cell cycle phases. These findings were validated by Western blotting, showing that levels of cyclin B1, the primary regulator of early events of mitosis, together with E2F2 and, to a lesser extent, cyclin D1/2, were lower in MPF-R treated with *RET*-siRNA compared to cells treated with control-siRNA (**Figure 3C**). Conversely, multiple regulators of G1-S phase transition, such as CDK4/6, cyclin D3, p-Rb, CDK2, and cyclin E, were found upregulated, likely to compensate for the cell division inhibition induced by RET knockdown. Together, our data suggest that RET is a driver of cell cycle progression at the G2-M phase of the cell cycle in combined CDK4/6i- and fulvestrant-resistant ER+ breast cancer cells.

### The RET-selective inhibitor selpercatinib inhibits growth of combined CDK4/6i- and fulvestrant-resistant ER+ breast cancer cells and CDK4/6i-resistant patient-derived organoids

**Figure 4:**
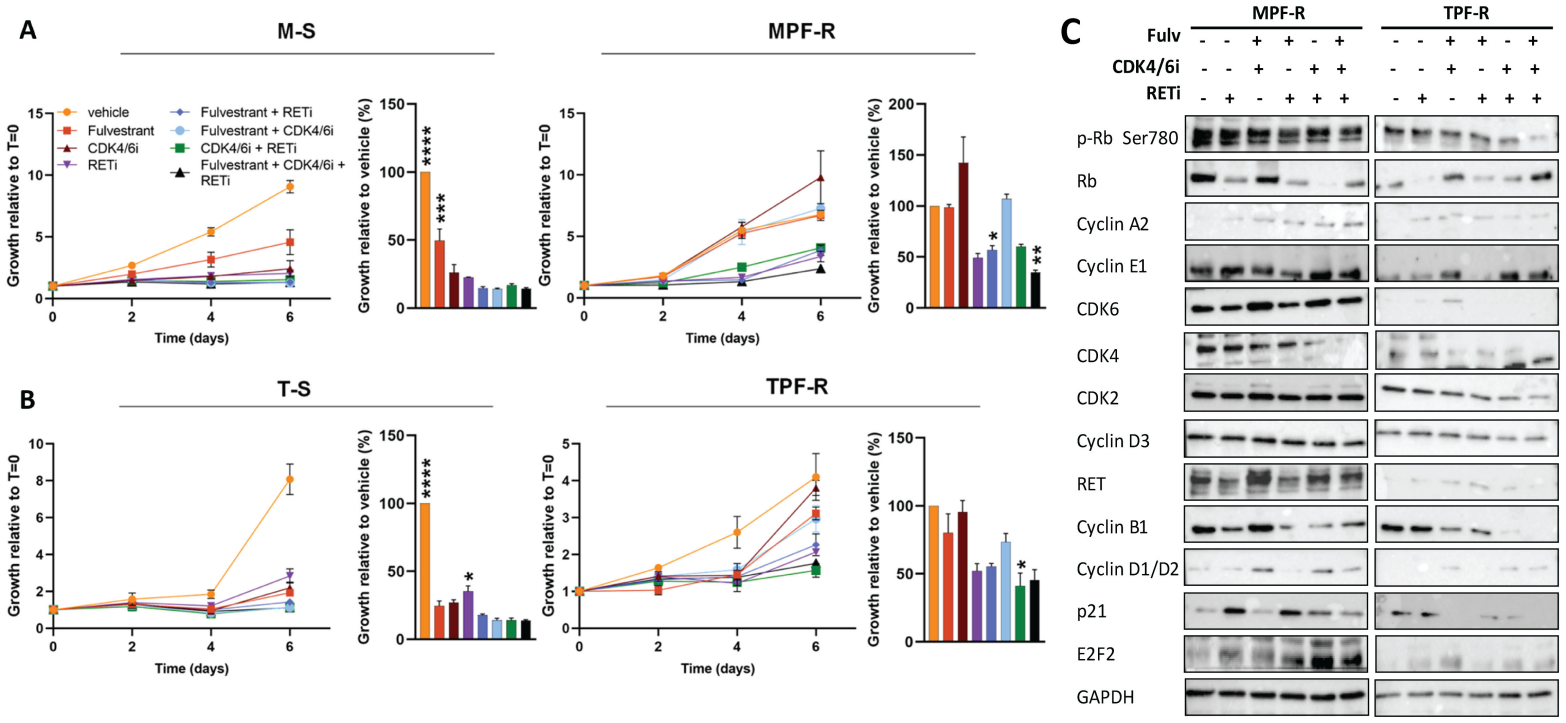
RETi resensitizes combined CDK4/6i- and fulvestrant-resistant ER+ breast cancer cells. Cell growth of **(A)** MPF-R and M-S cells and **(B)** TPF-R and T-S cells over six days in the presence of fulvestrant (100nM), CDK4/6i (200 nM) and RETi (5µM) alone or different combinations analyzed by crystal violet assay. Growth at day six is represented by columns. The data represents the mean of three biological replicates ± SEM. Asterisks indicate significant differences in one-way ANOVA tests at day six. Means are compared to the mean of the standard combined CDK4/6i and fulvestrant (*0.01 < p < 0.05, **0.001 < p < 0.01, ***0.0001 < p < 0.001, and ****p < 0.0001). **(C)** Western blotting of cell cycle regulators in MPF-R and TPF-R cells treated with RETi alone or combined with CDK4/6i and/or fulvestrant. GAPDH was used as a loading control.

To evaluate whether we could pharmacologically overcome resistance to combined CDK4/6i and fulvestrant, we examined the effect of the RET-selective inhibitor, selpercatinib, alone or in combination with CDK4/6i and/or fulvestrant, on the growth of MPF-R and TPF-R cells resistant to CDK4/6i and fulvestrant. Although the concentration of selpercatinib selected based on the IC50 calculation was high (5 µM), it remains within the maximum serum concentration observed in patients (mean steady-state Cmax 2980 ng/mL or 5.67 µM) (49). Treatment with selpercatinib resensitized resistant MPF-R and TPF-R cells to combined CDK4/6i and fulvestrant (**Figures 4A, B**). Indeed, incubation with the triple combination consisting of fulvestrant, CDK4/6i, and RETi significantly reduced the growth of MPF-R cells compared to combined CDK4/6i and fulvestrant. Furthermore, the triple combination more efficiently inhibited the growth of TPF-R cells compared to dual therapy with fulvestrant and RETi, although the difference did not reach statistical significance. Interestingly, the dual combination with RETi and either fulvestrant or CDK4/6i reduced MPF-R and TPF-R cells growth more efficiently than combined CDK4/6i and fulvestrant (**Figures 4A, B**). These data further indicate that ER+ breast cancer cells utilize RET upregulation to acquire resistance to combined CDK4/6i and endocrine therapy.

To validate these findings, we tested the efficacy of RETi in combination with fulvestrant with or without CDK4/6i in another combined CDK4/6i- and fulvestrant-resistant ER+ cell model derived from ZR-75-1 cells (**Supplementary Figures S1A, B**). Consistently with the findings in the other two models, we observed that all treatments significantly inhibited the growth of sensitive Z-S cells in comparison to the vehicle, while the growth of combined CDK4/6i- and fulvestrant-resistant ZPF-R cells was only significantly inhibited by the triple combination with fulvestrant, CDK4/6i and RETi (**Supplementary Figure S1B**). Furthermore, we investigated changes in cell cycle regulators upon treatment with the RETi selpercatinib in the three ER+ cell line models (**Figure 4C**; **Supplementary Figure 1C**). Treatment with selpercatinib induced marked reductions in cyclin B1 levels, a key regulator of early mitotic events, particularly when combined with a CDK4/6i. These findings parallel the trends observed following *RET* siRNA-mediated knockdown (**Figure 3C**). However, contrary to the upregulation of multiple modulators facilitating G1-S phase transition observed after *RET* knockdown, treatment with selpercatinib led to more efficient inhibition of these regulators, including CDK4, CDK6, cyclin D, and Rb, while leaving others unchanged (**Figure 4C**; **Supplementary Figure 1C**). Notably, the CDK inhibitor p21 showed a substantial increase following treatment with RETi. Collectively, these data underscore a blockade of both early and late cell cycle phase progression by RET inhibition with selpercatinib, thereby suggesting a more profound suppression of cell cycle activity.

**Figure 5:**
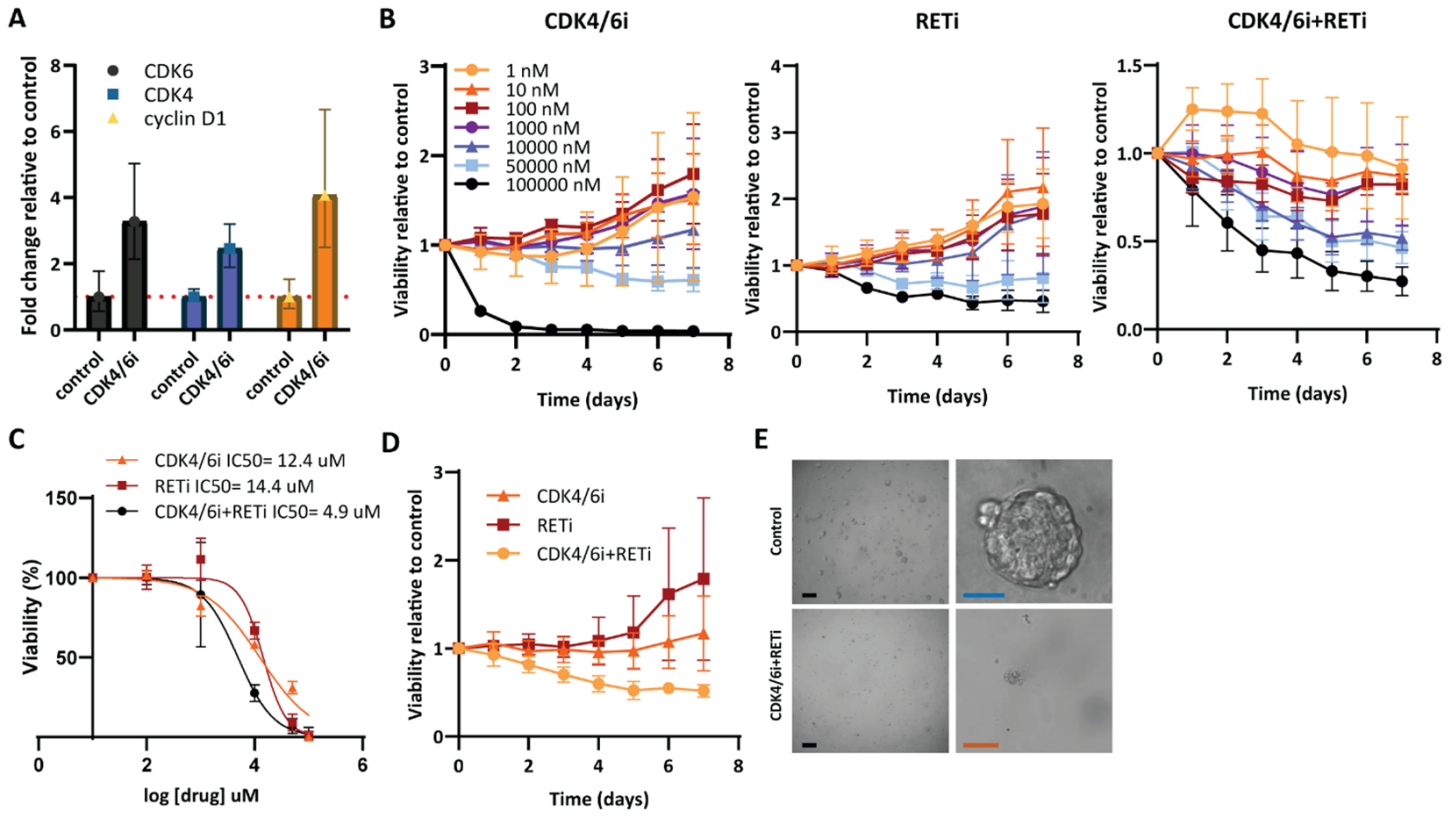
Combined CDK4/6i and RETi efficiently reduces viability of breast cancer patient-derived organoids resistant to CDK4/6i. **(A)** RT-qPCR verifying *CDK6*, *CDK4*, and *CCND1* gene expression alterations upon palbociclib treatment. The expression was normalized using the gene *PUM1* and shown as relative expression in control vs. treated with CDK4/6i. **(B)** Effect of increasing concentrations of CDK4/6i palbociclib and RETi selpercatinib, alone or RETi combined with a fixed concentration of CDK4/6i (1 µM), on the viability of patient-derived breast cancer organoid PDO-P48 for seven days. The results represent the mean ± SEMs of three replicates relative to the control (untreated). **(C)** Dose-effect curves of each single drug, CDK4/6i and RETi, or RETi combined with a fixed concentration of CDK4/6i (1 µM), during treatment of PDO-P48 for seven days. IC50 were calculated by normalizing the transformed data and using the non-linear curve fitting method “log(inhibitor) vs. normalized response – Variable slope”. **(D)** Viability of PDO-P48 treated with 10 µM of CDK4/6i and RETi single agents or their combination for seven days. **(E)** Brightfield images depicting PDO-P48 control (untreated) and treated with combined CDK4/6i and RETi. Scale bars: black 400 µm; blue 100 µm; orange 200 µm.

Next, we evaluated the efficacy of RETi selpercatinib and CDK4/6i palbociclib, alone or combined, in ER+ patient-derived breast cancer organoids (PDO-P48) that exhibited high IC50 towards CDK4/6i, and thus primary resistance to this treatment (**Figure 5**). This model exhibited increased *CDK4*, *CDK6*, and *CCND1* expression upon treatment with CDK4/6i (**Figure 5A**). Importantly, combined CDK4/6i and RETi markedly impaired PDO viability at lower concentrations (10 µM) compared to either CDK4/6i or RETi alone (50 and 100 µM, **Figure 5B**). Notably, the IC50 of combined CDK4/6i and RETi was lower than that of CDK4/6i alone in PDO-P48 (**Figure 5C**). Treatment of the organoids with 10 µM of CDK4/6i and RETi, combined or as single agents, showed that the combined treatment reduced cell viability relative to controls (untreated organoids), while CDK4/6i or RETi alone did not impair the organoid’s viability (**Figures 5D, E**). The combination of CDK4/6i and RETi demonstrated significant synergy within the concentration range 0.001-10 µM of RETi and 0.0001-1µM of CDK4/6i, as assessed using the Bliss model (**Supplementary Figure S2**; Mean synergy score: 22.85, *p*=3.91e-51). These data support the efficacy of combined CDK4/6i and RETi in tumors with poor response to CDK4/6i, such as PDO-P48.

### High expression of RET correlates with poor clinical outcomes in patients with ER+/HER2- breast cancer treated with endocrine therapy

**Figure 6:**
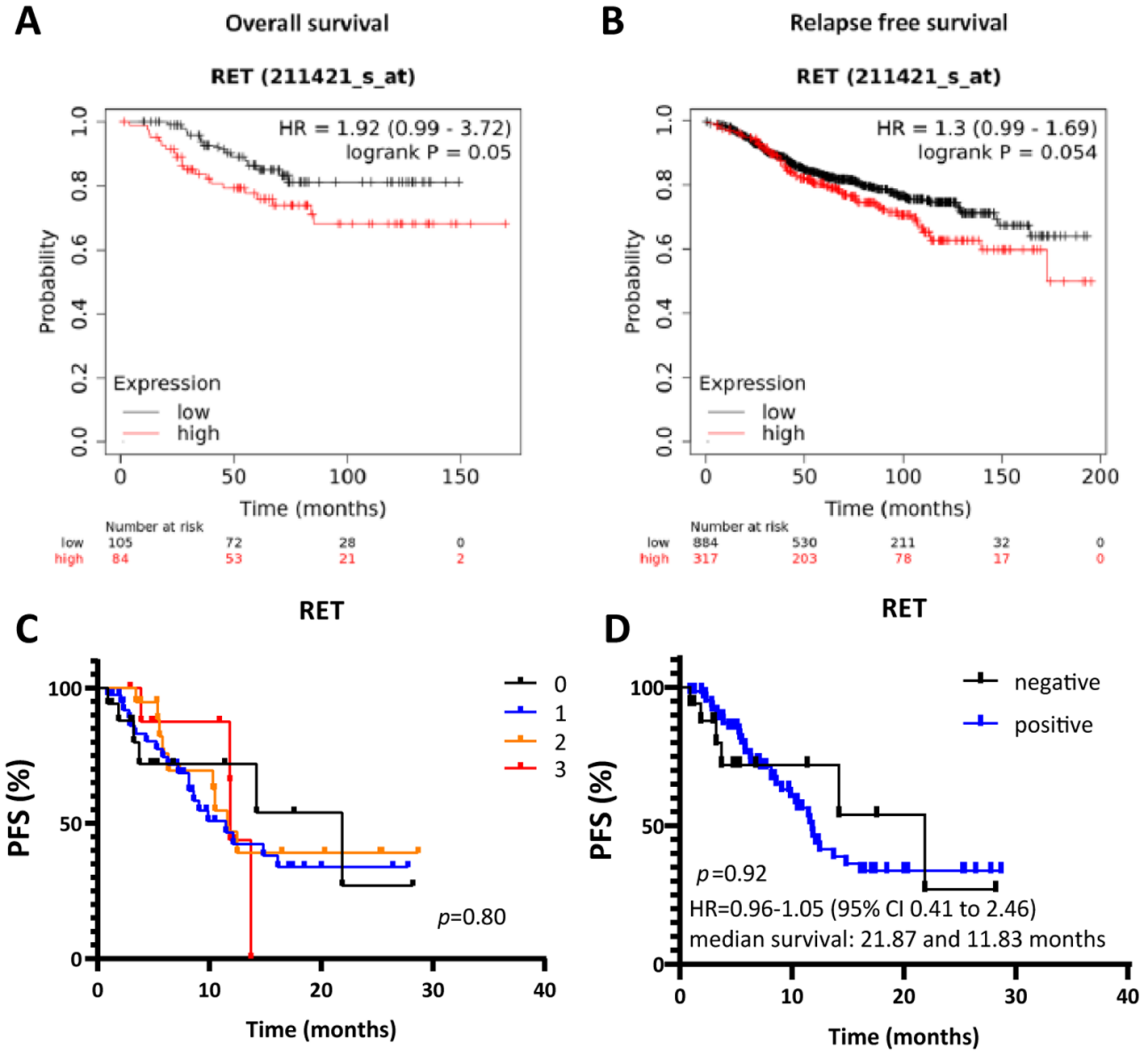
High RET expression correlates with shorter overall and relapse-free survival in patients with ER+ breast cancer receiving endocrine therapy. Kaplan-Meier survival curves for **(A)** OS and **(B)** RFS for RET expression by KM plotter analysis. **(C, D)** Kaplan-Meier survival curves evaluating PFS according to RET intensity score in ER+ metastatic lesions from patients treated with combined CDK4/6i and endocrine therapy. **(D)** Cut-off values: negative RET: intensity = 0; positive RET: intensity ≥ 1. A two-sided p-value calculated using Log-rank testing is shown.

Finally, we evaluated the clinical relevance of RET by assessing the correlation between RET expression and clinical outcomes in ER+ breast cancer patients. We first used the web-based tool Kaplan-Meier plotter (50) to assess the correlation between *RET* mRNA expression and overall survival (OS) and relapse-free survival (RFS) in a cohort of ER+ breast cancer patients receiving endocrine treatment in the primary setting. High *RET* mRNA expression significantly correlated with shorter OS (n=189, p=0.05, HR=1.92; **Figure 6A**) in ER+, HER2- breast cancer patients treated with endocrine therapy. The estimated 10-year survival was 70% for patients with high *RET* expression and 85% for patients with low *RET* expression (**Figure 6A**). High *RET* expression was also associated with shorter RFS (n=1201, p=0.054, HR=1.3; **Figure 6B**). The median time to relapse was 15 years (180 months) in the high-*RET* group, whereas the median time to progression in the low-*RET* group was 16 years (200 months) (**Figure 6B**). Multivariate analysis including *RET*, *MKi67*, and *ESR1* expression levels revealed that *MKi67* and *RET* expression are independent prognostic factors for RFS (HR 1.52, p = 0.0017; HR 1.34, p = 0.034, respectively) but not for OS (HR 1.14, p = 0.73; HR 1.89, p = 0.061, respectively; **Supplementary Table S2**).

Next, we evaluated the clinical relevance of RET as a biomarker of response/resistance to combined CDK4/6i and endocrine therapy in a cohort of ER+ advanced breast cancer patients that has been previously described (37, 48). The protein expression levels of RET were evaluated in whole sections of metastatic lesions before treatment initiation with combined CDK4/6i and endocrine therapy. RET score was determined on a semi-quantitative scale of 0-3, with 0 meaning no staining and 3 as the most intense staining (**Figure 6C**). Although the survival analysis indicated shorter median survival in the RET-positive (intensity ≥1) compared to RET-negative (intensity = 0) groups (11.83 and 21.87 months, respectively), differences in progression-free survival (PFS) between the two groups were not statistically significant (*p=*0.92, **Figure 6D**). Finally, we observed a higher mean of *RET* relative expression at baseline in the CDK4/6i-resistant compared to the -sensitive samples (1.08 and 0.93, respectively) in the neoadjuvant NeoPalAna trial (**Supplementary Figure S3**), which assessed the antiproliferative activity of the CDK4/6i palbociclib in breast cancer neoadjuvant setting (40).

## Discussion

Although combined CDK4/6i and endocrine therapy has significantly improved outcome of patients with advanced ER+ breast cancer, progression is expected and thus new therapeutic strategies to overcome treatment resistance are urgently needed. In this study, we show that RET is upregulated in breast cancer cell lines resistant to combined CDK4/6i and endocrine therapy compared to cells resistant to endocrine therapy alone, and inhibition of RET, either by siRNA-mediated knockdown or with the RET-selective inhibitor selpercatinib, alone or in combination with CDK4/6i and/or fulvestrant, reduced growth of CDK4/6i-resistant ER+ breast cancer cell lines and patient-derived organoids. In this study, we focused on the CDK4/6i palbociclib as it has been approved for a more extended period and is more frequently used in clinical practice. Nonetheless, we have previously demonstrated cross-resistance with ribociclib and abemaciclib in these cell models (37). Selpercatinib is approved for clinical use in patients with NSCLC, PTC, and MTC with *RET*-activating fusions or mutations. We examined our CDK4/6i- and fulvestrant-resistant cell lines for *RET* fusions using RNA-sequencing and *RET* mutations with panel NGS, but none were found. Since the drug has also shown efficacy on *RET*-wildtype tumors, it may also be useful in patients with RET overexpressing cancers. Indeed, our data support the addition of RETi to CDK4/6i and/or endocrine therapy as a therapeutic strategy following resistance to combined CDK4/6i and endocrine therapy in ER+ breast tumors exhibiting RET overexpression. Although previous studies have shown that the addition of the RETi pralsetinib to CDK4/6i further suppressed the growth of breast cancer cells with active *ESR1* fusions resistant to endocrine therapy compared to CDK4/6i alone (51), our study is the first, to our knowledge, to suggest an association between RET overexpression and resistance to combined CDK4/6i and endocrine therapy in ER+ breast cancer.

Previous studies have shown that RET induces estrogen-independent ERα phosphorylation and expression of ER target genes in ER+ breast cancer cells (17, 52). Overexpression of RET or its ligand GDNF has been associated with resistance to ER-targeted treatment (tamoxifen) through activation of the RAS/RAF/MEK/ERK or the mTOR/P70S6K pathways. This is consistent with our finding that RET induces growth and proliferation during combined CDK4/6i and endocrine therapy, though we have not evaluated alterations of PI3K and ERK as these pathways were not significantly altered following *RET* knockdown in cells resistant to combined CDK4/6i and fulvestrant. Importantly, we showed that *RET* is significantly overexpressed in CDK4/6i- and endocrine therapy-resistant compared to single endocrine therapy-resistant cells by global gene expression analysis and RNA sequencing, indicating that the observed *RET* upregulation is not a result of endocrine treatment alone. We further found that *RET* silencing using *RET*-specific siRNAs significantly inhibited the growth of MCF7-derived ER+ breast cancer cells resistant to combined CDK4/6i and endocrine therapy (MPF-R) but not of T47D-derived double-resistant cell line (TPF-R). Although TPF-R showed increased RET expression compared to the sensitive parental T-S cell line, the amount of RET was significantly lower than in MPF-R, both at mRNA and protein levels. The difference in the effect of *RET* silencing in the growth of the two CDK4/6i and endocrine therapy-resistant cell lines MPF-R and TPF-R might be due to different levels of activation of the PI3K/AKT/mTOR and RAS/RAF/MEK/ERK pathways in MCF-7 and T47D parental cells. Indeed, increased AKT and ERK activation has been observed in the MCF7-derived fulvestrant-resistant cell line (MF-R) due to its reliance on HER2 receptors for growth compared to the sensitive parental cell line, but this was not observed in the T47D-derived fulvestrant-resistant cell line (TF-R) (53–55). Since RET activates the RAS/MAPK and PI3K/AKT pathways in breast cancer cell lines, this might suggest that cell lines less dependent on these growth pathways will likely respond less to RET inhibition. Notably, RET inhibition with the approved inhibitor selpercatinib combined with CDK4/6i significantly impaired the growth of both MPF-R and TPF-R cell models, which suggests that additional or alternative growth pathways are impaired with RET-selective inhibitor compared to gene silencing. Furthermore, we observed that adding RETi was required to resensitize CDK4/6i-resistant patient-derived organoids. These data concur with a recent phase Ib/II clinical study of lenvatinib, a multikinase inhibitor with potent activity against RET, in patients with ER+ advanced breast cancer, which showed efficacy after progression on prior endocrine therapy with or without CDK4/6 inhibitor therapy (56).

Mechanistically, RET inhibition with selpercatinib was associated with a significant decrease in the activation of pathways involved in both the G1-S and G2-M phase transition of the cell cycle. This indicates that RET plays a role in multiple phases of the cell cycle, which has not been reported previously. Earlier studies have shown that RET upregulates the transcription of cyclin D1, leading to cell cycle progression at the G1-S phase and tamoxifen resistance, an effect blocked by the addition of a CDK4/6i (57). In our study, we observed that treatment with the RETi selpercatinib caused a marked decrease in cyclin D1, which regulates G1-S phase progression, and cyclin B1, which regulates the early stages of the M-phase of the cell cycle. Together, this suggests blockade of both early and late cell cycle phase progression by RET inhibition. RETi-induced downregulation of the cyclin D1-CDK4/6-Rb axis may lead to sustained hypophosphorylation of Rb, increasing its susceptibility to proteasome-mediated degradation (58). Although we have not investigated the mechanism driving the increased expression of RET in our cell models of resistance to combined CDK4/6i and endocrine therapy, we hypothesize that overexpression of cyclin D1 observed in the resistant cells (59) may promote the transcription of ER-mediated genes, such as *RET*. While we cannot exclude that the growth reduction observed in cells resistant to combined CDK4/6i and fulvestrant may be due to a general anti-proliferative effect of RET inhibition, we believe that the elevated levels of RET and its involvement in regulating both early and late cell cycle phase progression in these resistant cells indicate a more specific function related to CDK4/6i resistance. Finally, we show that high mRNA levels of *RET* significantly correlated with shorter OS and RFS in patients with primary ER+ breast cancer who received any type of endocrine therapy. These findings concur with other studies showing increased RET expression in primary ER+ breast cancer following adjuvant endocrine therapy and with the observation that RET plays a role in resistance to endocrine therapy (16, 17, 52). Indeed, we observed increased levels of RET in cells resistant to fulvestrant alone, but we showed further upregulation of RET following treatment with combined CDK4/6i and endocrine therapy compared to single endocrine therapy. Although we observed a shorter median survival to combined CDK4/6i and endocrine therapy in patients with RET-positive compared to RET-negative tumors, there was no significant correlation between RET protein expression and PFS in this patient cohort. These findings suggest that, although RET overexpression may be involved in the resistance mechanisms to combined CDK4/6i and endocrine therapy, it is likely not a robust biomarker of response to this treatment in metastatic samples obtained prior to treatment initiation. Future studies with larger clinical cohorts should address this. Importantly, the lack of association between RET level before treatment and PFS does not diminish the potential role of RET in the mechanisms of resistance to combined CDK4/6i and endocrine therapy, as supported by data from three ER+ cell models and a patient-derived organoid model resistant to CDK4/6i. Nevertheless, we underscore the preliminary nature of our findings and the need for further validation in additional clinical samples and patient-derived xenograft models.

## Conclusions

RET overexpression appears to contribute to resistance to combined CDK4/6i and endocrine therapy in ER+ breast cancer by promoting cell cycle progression at the mitotic phase. RET inhibition could be a potential treatment strategy for patients who develop resistance to combined CDK4/6i and endocrine therapy and with tumors exhibiting high RET expression.

## Data Availability

The generated gene expression and RNAseq data are available in the gene expression omnibus (GEO) database (GSE228637 and GSE268699). Data on survival analyses and immunohistochemical stainings are not publicly available to ensure patient privacy, but will be provided to authorized researchers with an approved Institutional Review Board application and approval from the Regional Committee on Health Research Ethics for Southern Denmark. For data access, please contact the corresponding author. All other datasets generated during the study will be available upon reasonable request to the corresponding author, Carla L. Alves, email address: calves@health.sdu.dk. Uncropped Western blots are included in the **Supplementary Material**.

## Glossary

AI: Aromatase Inhibitor
ANOVA: Analysis of variance
CDK4/6: Cyclin-dependent kinase 4 and 6
CDK4/6i: CDK4/6 inhibitor
DMEM/F-12: Dulbecco’s Modified Eagle Medium
DMSO: Dimethyl sulfoxide
EMA: European Medicines Agency
ER: Estrogen Receptor
ER+: Estrogen Receptor positive
ERK: Extracellular regulated kinase
ESR1: Gene encoding estrogen receptor
FBS: Fetal bovine serum
FDA: Food and Drug Administration
FDR: False discovery rate
FFPE: Formalin fixed paraffin embedded
GAPDH: Glyceraldehyde-3-phosphate dehydrogenase
GDNF: Glial cell line-derived neurotrophic factor
GFL: GDNF family of ligands
GFRα1-4: GDNF receptor 1-4
GSEA: Gene set enrichment analysis
HER2: Human epidermal growth receptor 2
HRP: Horseradish peroxidase
IHC: Immunohistochemistry
JAK: Janus Kinase
MAPK: Mitogen-activated protein kinase
MEK/MAPKK: Mitogen-activated protein kinase kinase
MTC: Medullary thyroid cancer
mTOR: Mammalian target of rapamycin
NGS: Next-generation sequencing
NSCLC: Non-small cell lung cancer
OS: Overall survival
PFS: Progression-Free Survival
PI3K: Phosphatidylinositol 3-kinase
PIK3CA: Phosphatidylinositol-4,5-bisphosphate 3-kinase catalytic subunit alpha
PTC: Papillary thyroid carcinoma
PUM1: Pumilio RNA Binding Family Member 1
PVDF: Polyvinylidene fluoride
Rb: Retinoblastoma
RET: Rearranged during transfection
RFS: Relapse-free survival
RPMI: Roswell Park Memorial Institute Medium
RT-qPCR: Quantitative real-time polymerase chain reaction
SDS-PAGE: Sodium dodecyl sulfate-polyacrylamide gel electrophoresis
SERD: Selective estrogen receptor degrader
siRNA: Small interfering RNA
STAT: Signal transducer and activator of transcription
