# Supplementary material for: RET inhibition overcomes resistance to combined CDK4/6 inhibitor and endocrine therapy in ER+ breast cancer
Source: Front Oncol. 2025 Jan 27;14:1497093. doi: 10.3389/fonc.2024.1497093 (PMC11808005; doi:10.3389/fonc.2024.1497093)
Supplement: Supplementary file 2 [file DataSheet1.docx]

**Supplementary Table S2**. Multivariate analysis of RFS and OS according to *MKI67*, *ESR1* and *RET* expression in ER+/HER2- breast tumors treated with endocrine therapy.

| **Variable** | **RFS HR (95% CI)** | ^1^ **RFS *p*** | **OS HR (95% CI)** | ^1^ **OS *p*** |  |  |  |  |  |  |  |  |  |  |  |  |  |  |  |  |  |  |  |  |
| --- | --- | --- | --- | --- | --- | --- | --- | --- | --- | --- | --- | --- | --- | --- | --- | --- | --- | --- | --- | --- | --- | --- | --- | --- |
| *MKI67* | 1.52 (1.17 - 1.98) | 0.0017** | 1.14 (0.54 - 2.43) | 0.730 |  |  |  |  |  |  |  |  |  |  |  |  |  |  |  |  |  |  |  |  |
| *ESR1* | 0.75 (0.33 – 1.72) | 0.5 | 2813933.01 (0 - inf) | 0.997 |  |  |  |  |  |  |  |  |  |  |  |  |  |  |  |  |  |  |  |  |
| *RET* | 1.34 (1.02 – 1.74) | 0.0339* | 1.89 (0.97 – 3.66) | 0.061 |  |  |  |  |  |  |  |  |  |  |  |  |  |  |  |  |  |  |  |  |

^1^ RFS: Relapse-free survival; OS: Overall survival. * p ≤ 0.05 and ** p ≤ 0.01

**Supplementary Figures**


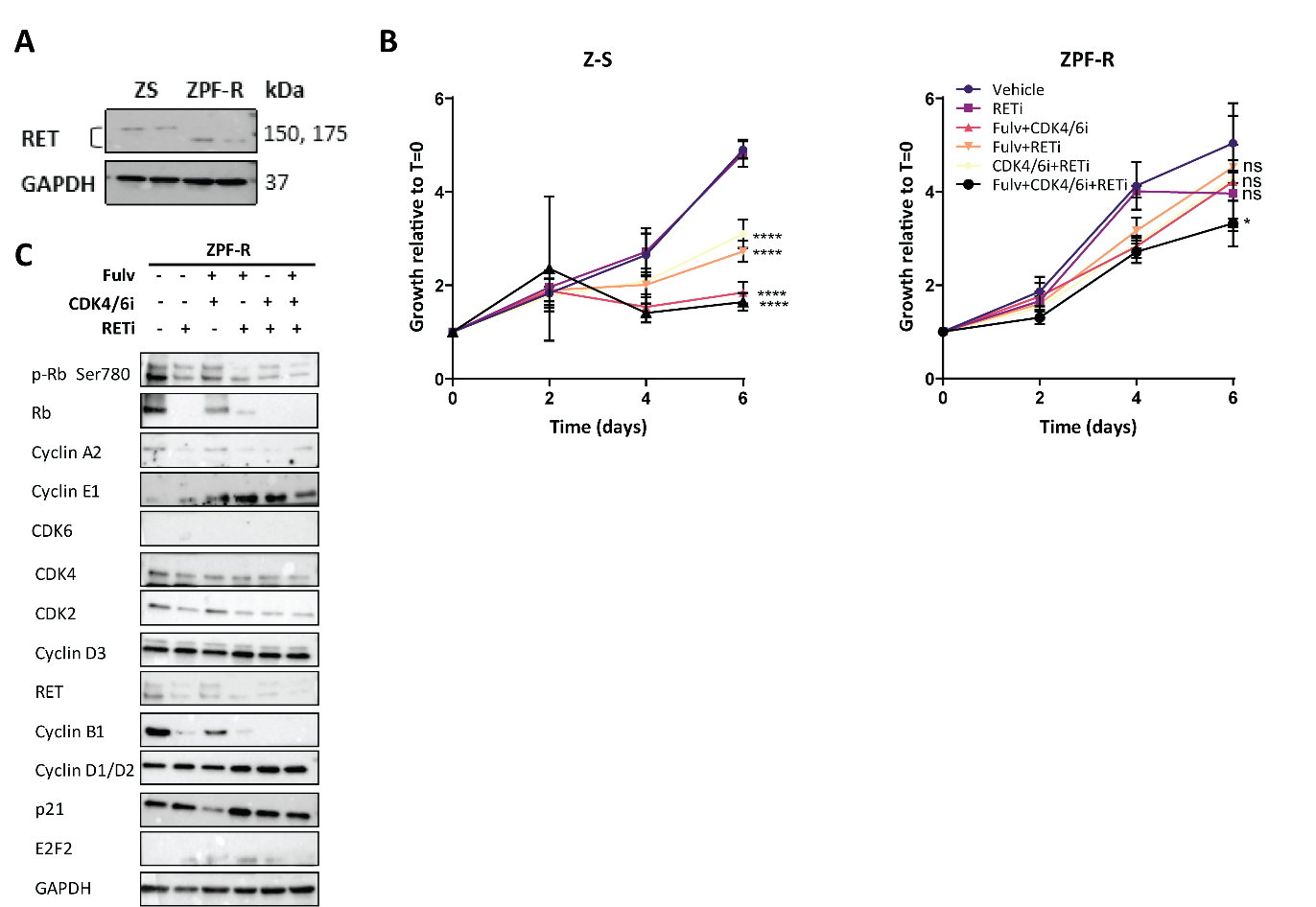


**Supplementary Figure S1. RETi inhibits growth of combined CDK4/6i- and fulvestrant-resistant ER+ breast cancer cells. A)** Western blotting analysis of RET expression in lysates from Z-S and ZPF-R cells. GAPDH was used as loading control. **B)** Cell growth over six days in the presence of fulvestrant (100nM), CDK4/6i (200 nM) and RETi (5µM) alone or different combinations analyzed by crystal violet assay. **C)** Western blotting of cell cycle regulators in ZPF-R cells treated with RETi alone or combined with CDK4/6i and/or fulvestrant. GAPDH was used as loading control.


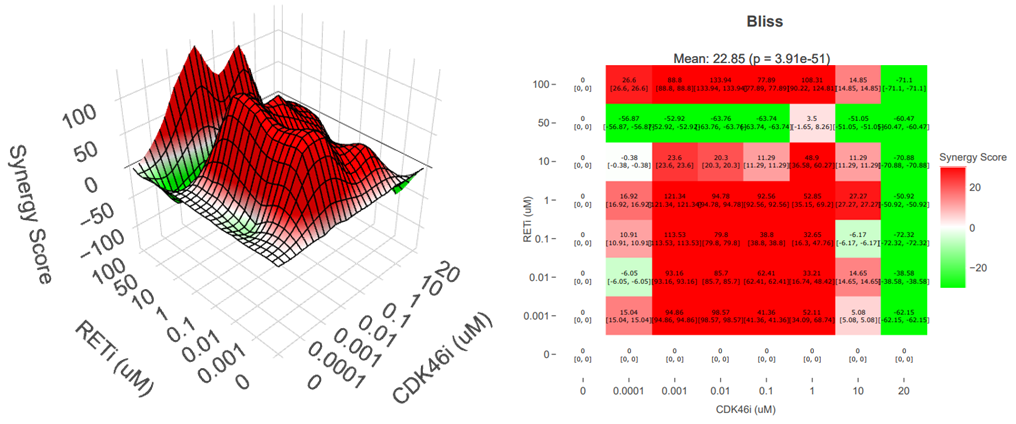


**Supplementary Figure S2. CDK4/6i and RETi exhibit synergistic effects in patient-derived organoids resistant to CDK4/6i**. Organoids were seeded in 96-well plates at a concentration of 200 organoids/µL in Cultrex (50 µL/well), followed by treatment with serially diluted CDK4/6i palbociclib or RETi selpercatinib alone, or in combination, at different concentrations for 7 days. Cell viability was evaluated with RealTime-Glo™ MT cell viability assay and Bliss synergy score was calculated using SynergyFinder.


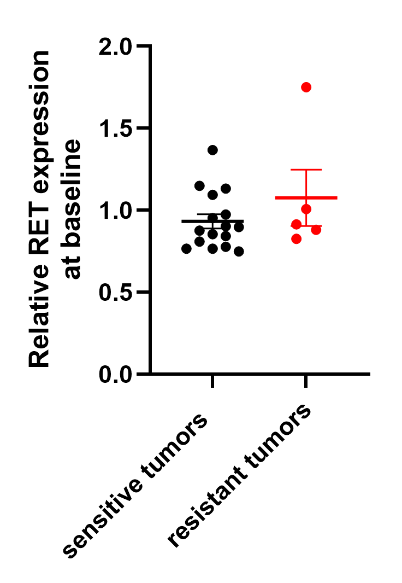


**Supplementary Figure S3. RET expression is increased in baseline samples from resistant- compared sensitive-CDK4/6i patients in the neoadjuvant NeoPalAna trial.** Relative expression of *RET* in baseline samples from primary breast cancer patients enrolled in the NeoPalAna trial. Data representing mean ± SEM of relative microarray reads are shown.


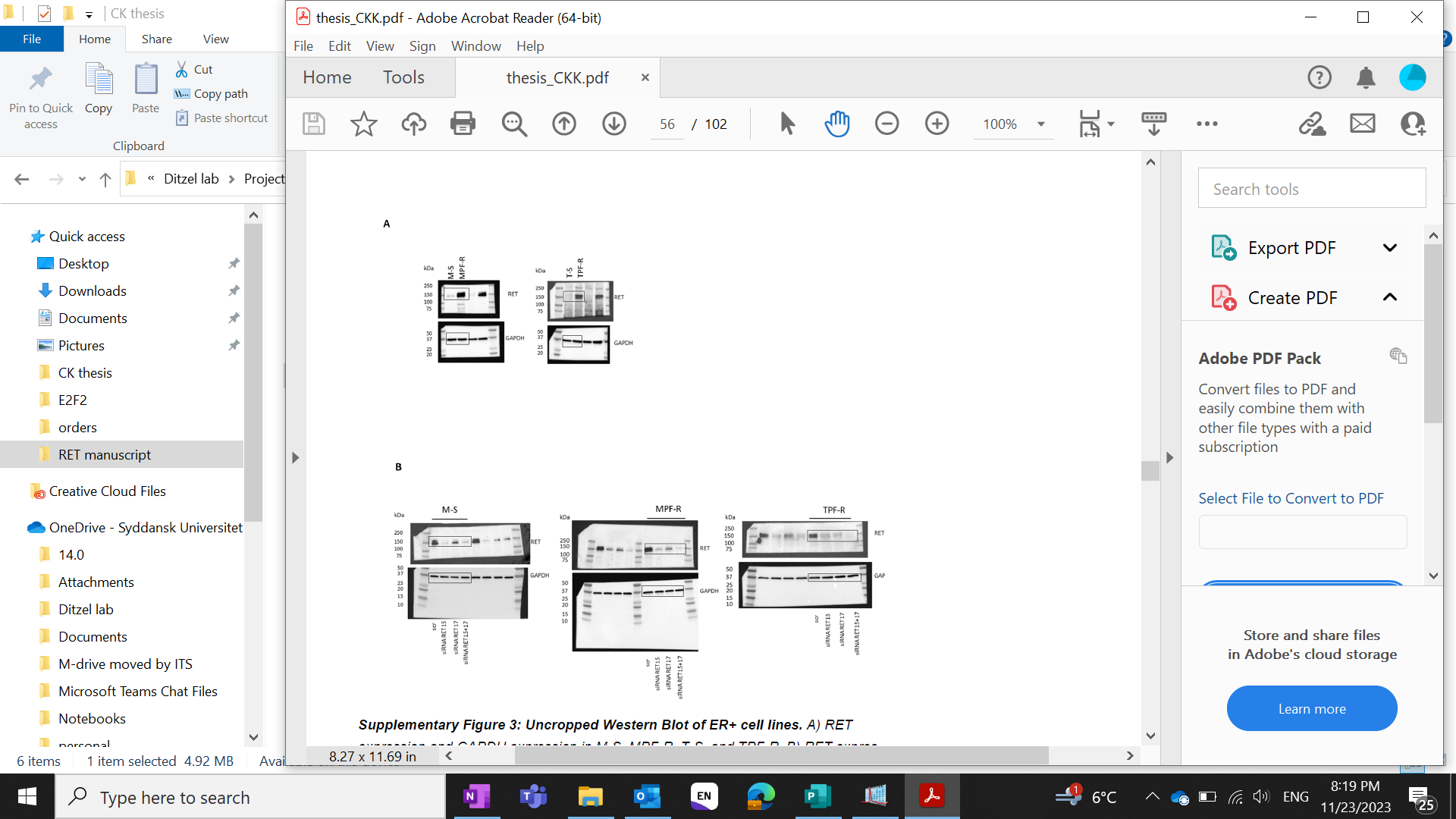
**Supplementary Figure S4. Uncropped Western blots of ER+ cell lines.** A) RET expression and GAPDH expression in M-S, MPF-R, T-S, and TPF-R. 10 µ and 50 µg of total protein of M-S/MPF-R and T-S/TPF-R, respectively, were loaded. B) RET expression following silencing of RET in M-S, MPF-R and TPF-R. siRNAs: scr: unspecific control siRNA, RET15 and RET17: RET-specific siRNAs.


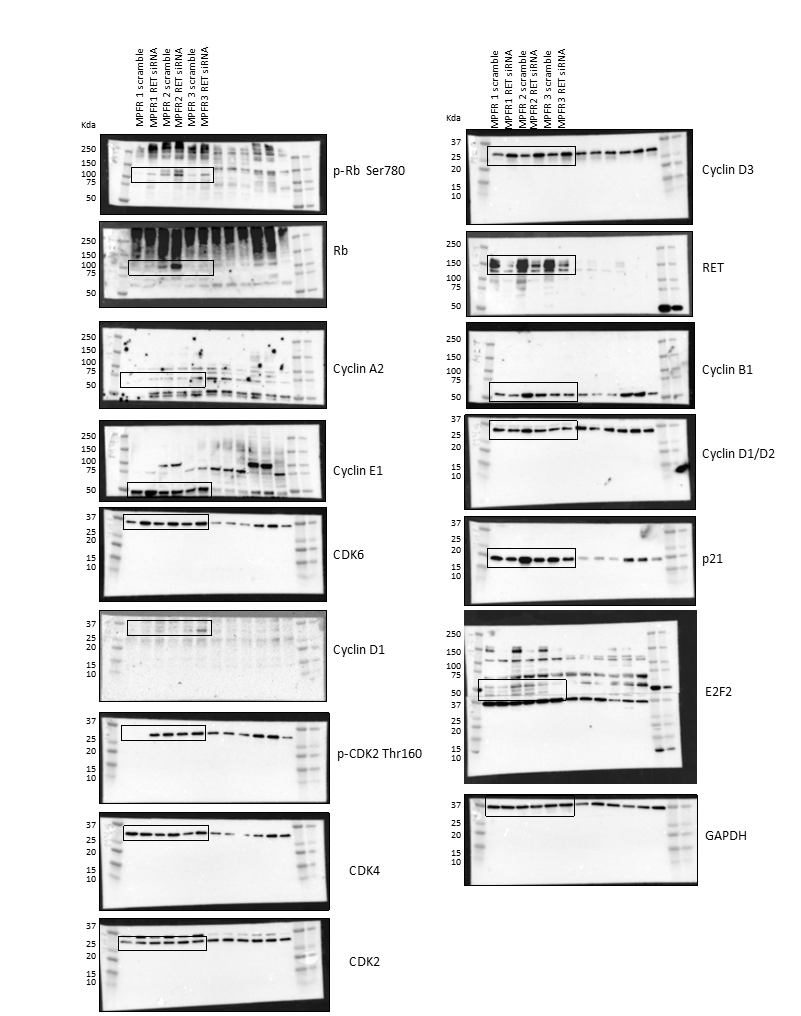


**Supplementary Figure S5: Uncropped Western blots of MPF-R cell lines treated with control (scramble) or *RET*-siRNA (pool of RET15 and RET17 siRNAs).** Expression of cell cycle regulators following silencing of *RET* in three biological replicates of MPF-R cells compared to controls.
